# The Experience of Novelty and the Novelty of Experience

**DOI:** 10.3389/fpsyg.2020.00322

**Published:** 2020-02-26

**Authors:** Liubov Skavronskaya, Brent Moyle, Noel Scott

**Affiliations:** ^1^USC Business, Faculty of Arts, Business and Law, University of the Sunshine Coast, Maroochydore, QLD, Australia; ^2^Department of Tourism, Sport and Hotel Management, Griffith University, Brisbane, QLD, Australia; ^3^Sustainability Research Centre, University of the Sunshine Coast, Sippy Downs, QLD, Australia

**Keywords:** novelty, tourism experience, cognitive appraisal theory, emotions, memory, psychology

## Abstract

In cognitive psychology novelty is an antecedent of attention, emotion, memory, and behavior. However, the relationship between novelty and experience memorability remains conceptually underdeveloped in tourism. This research applies cognitive appraisal theory (CAT) to explore the contribution of novelty and emotion to memorable tourism experiences (MTEs). Seventy-five novel travel episodes were identified through semi-structured interviews. Analysis focused on the antecedent and consequent conditions of novelty. Novel experiences, whether positive or negative, were identified as critical to experience memorability. Novelty could be segmented into trip-related and event-related dimensions. Novelty contributes to how spatial, temporal, and contextual details of tourism experiences are remembered and reconstructed due to the elicitation of intense emotions. Analysis revealed negative experiences deemed as novel were found to be re-evaluated and often remembered as a positive experience. A conceptual model titled “cognitive appraisal of novelty in memorable tourism experiences” is presented for consideration in future research. By applying a retrospective and prospective approach the conceptual model explores the role of novelty through the process of cognitive appraisal, identifying goals, attention, and prior experiences as central for the experience of novelty. Future research should consider the application of recent advance in CAT to advance inquiry on tourism experiences as a psychological phenomenon.

## Introduction

Experiences are an area of strong academic and practical interest (Kim, 2010; Tung and Ritchie, 2011a; Volo, 2013; Kirillova et al., 2017). Tourists' seek memorable tourism experiences (MTEs) (Scott et al., 2017), which have the propensity to generate destination loyalty (Chen and Rahman, 2018; Zhang H. et al., 2018), increase satisfaction (Kim, 2018) and promote emotional engagement (Michalkó et al., 2015). However, the creation of memorable experiences requires an understanding of the mental processes which occur at different stages of a tourist experience, including the antecedent and consequent conditions (Knobloch et al., 2017).

Previous studies of MTEs have focused on concepts such as motivation (Gnoth and Matteucci, 2014; Prayag et al., 2017; Yoo et al., 2018; Passafaro, 2019), expectations and satisfaction (Tynan and McKechnie, 2009; Kim, 2018), well-being and quality of life (Uysal et al., 2012), as well as emotions and memories (Chandralal and Valenzuela, 2015; Moyle et al., 2019). Psychological antecedents of MTEs include hedonism, involvement, knowledge, refreshment, meaningfulness, and novelty (Chandralal and Valenzuela, 2013; Kim, 2014). Recent studies indicate the importance of novelty in elicitation of emotions and a connection to memorability (Ma et al., 2017; Mitas and Bastiaansen, 2018). Although the importance of novelty has been established, there is a dearth of research on the relationship between novelty and MTEs.

A plausible reason for this discrepancy may be the dominance of behavioral rather than cognitive psychology in previous studies on tourism experiences (Skavronskaya et al., 2017a,b). Studies have found that emotions are related to increased memorability of a tourism experiences (Bastiaansen et al., 2019; Hosany et al., 2019), but do not provide adequate insights into the theoretical mechanisms by which these concepts are connected (Scott, 2020). Cognitive psychology is the field that examines the mechanisms by which our brain experiences and interprets external stimuli and provides insights into the mental processes connecting perception of stimuli with behavior (Neisser, 2014). Despite its relevance few studies have applied cognitive psychology to research on tourism experiences (Manthiou et al., 2014; Chandralal and Valenzuela, 2015).

**Figure 1:**
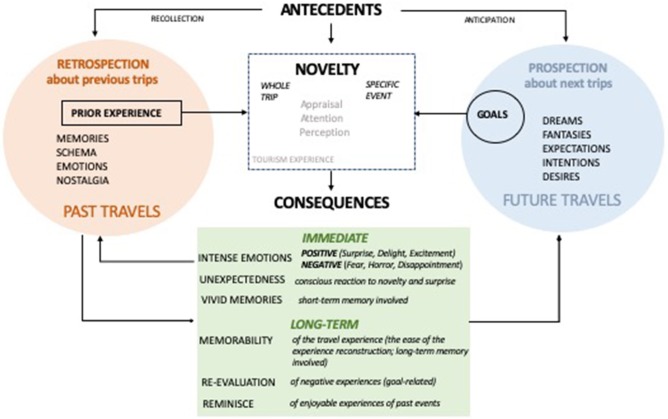
Cognitive appraisal of novelty in MTEs.

This research applies cognitive appraisal theory (CAT) (Arnold, 1960; Frijda et al., 1989; Lazarus, 1991; Roseman, 2013) to explore the intricate connection between novelty and the memorability of tourism experiences in order to add value to the extant behavioral studies on the tourism experience phenomenon. Semi-structured interviews with 25 respondents were used to identify 75 unforgettable travel experiences involving novelty. Analysis of the results provides a conceptual cognitive appraisal model of the role of novelty in MTEs (Figure 1). The model explores the influence of novelty in the formation of MTEs through the process of cognitive appraisal. Connected to this, the intersection between novelty with cognitive processes such as emotions, goals, attention, expectations, prior experiences (memory), and fantasies (prospection) is explored.

## Cognitive Psychology and Tourism Research

Tourism is an applied field that adopts theory from parent disciplines, including psychology (Weiler et al., 2012, 2018; Ruhanen et al., 2015). Most psychological theory applied in tourism is from the behavioral or socio-psychological subfields of psychology. The subfield of cognitive psychology has potential to advance conceptual understanding of the phenomenon of tourism, specifically the mental processes which underpin memorable experiences (Skavronskaya et al., 2017a). Cognitive psychology is focused on understanding the mechanisms by which our brain experiences and interprets the world (Scott, 2020).

Traditional cognitive psychology is based on an information processing or input-output model of the brain but focuses on the mental processes that connect perception of stimuli and subsequent behavior (Neisser, 1967). Cognition refers to all processes by which the sensory input is transformed, reduced, elaborated, stored, recovered, and used (Lachman et al., 2015) including attention, learning, consciousness, memory, and emotion (Neisser, 2014). The application of cognitive psychology theory to tourism experiences is an emerging area of research (Larsen, 2007; Agapito et al., 2013; Skavronskaya et al., 2017a; Campos et al., 2018; Scott, 2020).

For instance, the concept of attention may be used to better understand co-creation and mindful tourist experiences (Campos et al., 2016; Chen et al., 2017). The emotion of delight is elicited through high levels of the cognitive appraisal dimensions of goal interest and importance (Ma et al., 2017). The concept of a memory schema can help to study destination image change through manipulation of schema congruity (Zhang R. et al., 2018). Due to the dominance of behavioral paradigm in psychological studies on the tourism experiences, the literature considering experiences as a cognitive process are scarced. Therefore, CAT of emotions elicitation presenting a prominent theory with a capacity to add value to extant tourism experiences research. This research seeks to explore the cognitive process that connects novelty through emotion and memorability drawing on CAT (Arnold, 1960; Lazarus, 1991; Roseman, 2013).

## Cognitive Appraisal Theory

Following the cognitive processes conceptualized in CAT emotions are elicited after an individual evaluates their experience on a limited number of appraisal dimensions relevant to their personal goals at the time of the experience (Bagozzi et al., 1999; Roseman, 2001; Johnson and Stewart, 2005). Johnson and Stewart (2005) define an appraisal as the “*implications of the situation for the interests and goals of the individual, and thereby determine the form that emotional reaction takes in a given situation*” (p. 4). An appraisal process is defined as evaluation of an event in the environment (Oatley and Johnson-Laird, 2014). In cognitive psychology CAT is often applied in the context of negative emotions, such as fear or anxiety (So et al., 2016) but more recently to positive emotions (Ma et al., 2017; Manthiou et al., 2017).

An emotional response is a reaction to the appraisal of an event in terms of the positive or negative fit to a goal or plan based on a limited number of appraisal dimensions (Massara et al., 2010). Key cognitive appraisal dimensions include outcome desirability or goal congruency, agency, certainty, attention, novelty, unexpectedness, and coping potential (Watson and Spence, 2007). CAT emphasizes the importance of a person's interpretation of an event, rather than the event itself, in determining emotional response. CAT proposes a sequential mechanism consisting of perception of a particular situation (experience), followed by an evaluation (appraisal) based on appraisal dimensions, from which a particular emotion is elicited with the potential to influence behavior (action) (Cassidy, 2013). Importantly, the emotional appraisal of a particular situation may or may not stimulate a behavioral outcome (Roseman, 2013).

A number of appraisal dimensions such as novelty, goal congruence, goal realization, and goal relevance have been identified (Ma et al., 2013). Manthiou et al. (2017) used dimensions of goal congruence and certainty to determine emotional valence of experiences on a luxury cruise. Hosany (2012) found the dimensions of goal congruence, pleasantness, and internal self-compatibility influence elicitation of joy, love, and positive surprise. Novelty is an important appraisal dimension in CAT and higher novelty is related to higher emotional arousal (Ma et al., 2013). The present research applies CAT to study how the novelty of an experience influences emotional responses that in turn enhance memory of an event.

## Defining Novelty

Novelty is a process of experiencing or encountering something different to the objects regularly encountered (Barto et al., 2013). In our daily routine, the words “*new*” or “*unusual*” are the most common synonyms of novelty. Novelty is a complex and subjective psychological concept (Mandler, 1997) with debate about its precise definition (Skavronskaya et al., 2019a).

Novelty has been defined as the extent to which a stimulus is discrepant or familiar for the individual compared to the typical information that a person is possesses (Cohen, 1993; Mak, 2015), or between what is known and what is discovered (Mather, 2013). In neuroscience, novelty is considered as a variable associated with activity in response to stimulation (Cloninger et al., 1994). Novelty is found to increase attention to a focal stimuli, promote and encode memory and modify goal-directed behavior (Bunzeck et al., 2012). Novel stimuli are found in a wide variety of environments and detection of novelty plays a key role in learning through redirection of attention to unknown and potentially important phenomena (Goldberg, 1994). It is also important for cognitive development and performance (Tournier et al., 2012).

In tourism, novelty has been defined as the “e*xtent to which an experience departs from an individual's expectation*” (Ma, 2013, p. 54). Novel tourism experiences are associated with unexpectedness, delight, surprise, thrill, and enjoyment (Ortony et al., 1988; Scherer, 1993; Roseman et al., 1996; Mitas and Bastiaansen, 2018). Novelty is considered as a motivational precursor of behavior (Berlyne, 1960, 1970; Dunman and Mattila, 2005), and associated with varied, complex, and intense feelings with a potential to achieve peak experiences (Cloninger et al., 1994).

The related concept of novelty-seeking is used to understand consumer behavior (Farias et al., 2014), tourists' typology (Cohen, 1972), and travel motivations (Dann, 1977; Crompton, 1979; Chon, 1989). Novelty-seeking is central in the understanding of tourists' destination choice (Petrick, 2002) and motivation (Dann, 1977; Crompton, 1979; Chon, 1989; Lee and Crompton, 1992). Novelty-seeking characterized by search for novel, varied, complex, and intense feelings, and a readiness to take physical, social, legal, and financial responsibility to achieve novel experiences (Cloninger et al., 1994).

Novelty is considered to be intricately connected to the experience of pleasure through experiences of flow, mindfulness, and creativity (Csikszentmihalyi, 1997; Filep et al., 2016). Novelty is related to emotional arousal through the alleviation of boredom and hedonic and eudemonic well-being (Iso-Ahola and Weissinger, 1990; Filep and Laing, 2019; Vada et al., 2019).

## Novelty Experience, Emotion, and Memory

Novelty is fundamental to tourism and travel (Mitas and Bastiaansen, 2018), and critical for MTEs (Kim J. H et al., 2012). Novelty in a pleasurable tourism experience is associated with suddenness or unexpectedness and with emotions such delight, surprise, and enjoyment (Ortony et al., 1988; Scherer, 1993; Roseman et al., 1996). Emotions are considered as critical for the delivery of MTEs (Bastiaansen et al., 2019; Skavronskaya et al., 2019b). Strong emotions resulting from a novel experience result in vivid memories, created through the secretion of chemicals including dopamine (Moyle et al., 2019). Recent studies on psychological antecedents of MTEs acknowledge novelty as one of the main dimensions underlying experience memorability (Chandralal and Valenzuela, 2013; Kim, 2014).

While the relationship between novel experiences, emotion, and memory has been previously discussed, the cognitive processes by which these concepts are related require further exploration (Tung and Ritchie, 2011a; Scott, 2020). There is need to explore the process by which novelty, memory and emotions are connected in the context of MTEs from cognitive psychology perspective due to dominance of behavioral paradigm in extant tourism experiences research. Furthermore, recent tourism literature has a tendency to focus predominantly on the antecedents of positive memorable experiences due to the hedonic nature of tourism (Tung and Ritchie, 2011a). However, the neuroscience literature indicates that novelty is independent from emotional valence (Förster et al., 2010), suggesting novelty is connected with both positive and negative emotions in MTEs.

Overall, novelty is a complex and subjective phenomenon fundamental for tourism (Mitas and Bastiaansen, 2018) with lack of comprehensive, structured theory (Witt, 2009a,b). How novelty connects with emotions and memories is not well-explored in tourism (Skavronskaya et al., 2019b). Thus, the present research draws on cognitive psychology to explore the connection between novelty and experience memorability in tourism.

## Method

Analysis of existing studies revealed quantitative methods, often using experimental approaches, dominated conceptually related studies in psychology and tourism (Li et al., 2015; Veal, 2017; Weiler et al., 2018). Furthermore, extant quantitative studies on MTEs predominantly focus on correlations between external tourism stimuli with consequent conditions, such as emotions or behavioral intentions, assuming that external stimuli have direct effect on outcome. The prevalence of quantitative studies in the tourism field is further demonstrated by studies on the antecedents and consequences of MTEs (Kim, 2014; Chandralal and Valenzuela, 2015; Coudounaris and Sthapit, 2017; Chen and Rahman, 2018). Results confirm the importance of novelty for eliciting emotions in tourism (Mitas and Bastiaansen, 2018). Therefore, quantitative studies on MTEs acknowledge various components in the process of cognitive appraisal as mediating factors connecting tourism stimuli with behavior (Ma et al., 2013, 2017; Campos et al., 2016; Kim, 2018).

Although drawing on cognitive psychology the results of these studies are also often interpreted in behavioral terms (Mari and Poggesi, 2013; Manthiou et al., 2017). Consequently, while informed by theory grounded in cognitive psychology, analysis is yet to explore, in-depth, how the process of cognitive appraisal contributes to MTEs in tourism. Exploring novelty through the process of cognitive appraisal has the potential to provide in-depth insights to further inform quantitative studies by enriching conceptual understanding of the mental processes which underpin experience memorability. Subsequently, this research utilized qualitative approach to study novelty in MTEs designed to advance conceptual understanding of the tourism experience as a psychological phenomenon, as suggested by previous studies (Tung and Ritchie, 2011a; Coelho et al., 2018). Therefore, to address the outlined gap, this research is applying qualitative approach with CAT as a conceptual framework to study MTEs and merging tourism with an area of cognitive psychology where quantitative methods dominate existing scholarly inquiry. Studying MTEs with CAT as a theoretical framework provide additional in-depth perspective on an organism mental state prior—during and after traveling, that quantitative studies are not able to cover. Thus, qualitative studies on the antecedent conditions of mental processes occurring prior and during the tourism experiences will help to better understand tourist behavior.

In-depth semi structured interviews were undertaken with 25 participants that were asked to recall three episodes of unforgettable and unexpected travel experiences. As a result, 75 episodes of memorable and novel travel experiences were collected and identified. Interviews were conducted in Russian in Saint Petersburg, Krasnodar, and Moscow between April and May 2018. Each interview took place in a public setting, usually, a café, with the duration of each interview between 30 and 60 min. Respondents were selected using convenience sampling (Ali et al., 2014). This sampling method is suitable as the core goal of the research is not generalizability, rather an in-depth exploration of novelty and memorability.

Interview questions designed utilizing core tenants from CAT depicted in previous studies focused on tourism experiences (Hosany, 2012; Ma et al., 2013; Manthiou et al., 2017). Probing questions explicitly designed to unearth the mental process that intricately connects novelty with experience memorability through emotions (See Table 1). Pilot interviews with two participants was applied to ensure the questions were appropriately structured and framed, resulting in minor modifications to the instrument prior to final launch. Particular attention was given to how novel experiences resulted in emotional responses and memorability. Episodes of novelty episodes were identified utilizing the criteria of a “*first time”* occurrence of a particular event or experience during traveling and a mismatch between expectations and actual experience (Meyer et al., 1991).

**Table 1:** Interview questions.

	**Dimension**	**Question(s)**
1	Prior experience	• Do you enjoy traveling? • Could you describe your travel history to me? • What was three most unforgettable trip you've had in your life? • Why do you think you still remember these trips?
2	Novelty	• Can you describe something you never experienced before in these unforgettable trips?
3	Goal	• Do you want it to happen? • Why it was important to you? • What was the purpose of that trips?
4	Surprise/Unexpectedness	• Can you describe your expectations? • What was the most unexpected/surprising during your trip?
5	Emotional response	• How did you feel before and after these trips?

Interviews were conducted in Russian, the participants' native language, to develop rapport with respondents who encouraged to speak openly and in a relaxed and engaged manner (Welch and Piekkari, 2006). The interview processes allowed the researcher to understand the in-depth meanings expressed by participants, as well as their cultural and social background (Chen et al., 2017). Interviews were recorded using a digital voice recorder, specifically a “Dictaphone” app, after the permission from each participant had been granted. Pseudonyms are used to preserve the anonymity of respondents. Table 2 shows the characteristics of respondents. Prior to analysis, all interviews were transcribed. Following Hall (2004), to improve the credibility and trustworthiness of the findings the transcripts from the interviews were double-blind translated to English and compared.

**Table 2:** Participants' profiles.

	**Pseudonym(s)**	**Age**	**Gender**	**Education level**	**Occupation**
1	RES_1	32	F	Doctor	Education
2	RES_2	33	F	Doctor	Research
3	RES_3	28	F	Degree	Retail
4	RES_4	30	F	Degree	Beauty consultant
5	RES_5	31	M	Doctor	Education
6	RES_6	28	M	Certificate	Trainer
7	RES_7	26	F	Degree	Student
8	RES_8	32	F	Degree	Education
9	RES_9	28	F	Certificate	Education
10	RES_10	29	F	Doctor	Education
11	RES_11	30	F	Degree	Filmmaker
12	RES_12	32	F	Degree	Translator
13	RES_13	32	F	Degree	Manager
14	RES_14	28	F	Doctor	Education
15	RES_15	26	F	Degree	Engineer
16	RES_16	29	F	Degree	Student
17	RES_17	34	F	Doctor	Education
18	RES_18	30	F	Degree	Entrepreneur
19	RES_19	28	M	Degree	Designer
20	RES_20	34	F	Degree	Retail
21	RES_21	28	F	Degree	Retail
22	RES_22	29	M	Degree	Aviation
23	RES_23	38	F	Master	Designer
24	RES_24	35	M	Master	Entrepreneur
25	RES_25	31	M	Degree	Auditor

CAT provided a theoretical structure to the enquiry and a grounded theory approach was used for analysis, specifically the coding and interpretation of the data (Corbin and Strauss, 1990; Matteucci and Gnoth, 2017). Following Sharma and Sarmah (2019), this study applied NVivo 12 to increase the efficiency of data analysis. Interviews were coded for themes, using open, axial, and selective coding as previously applied in Kennelly et al. (2013); Hillman et al. (2018), and Moyle et al. (2018).

Open coding enabled to identify and record the common emergent themes, such as the episodes of novelty, surprise, and unexpectedness in positively and negatively remembered tourism experiences. Axial coding ensured that common emergent themes has been grouped into categories such as antecedent and consequent conditions of novelty in the context of tourism experiences. An interceder reliability check was performed on the emergent themes from two coders independent to the research, resulting in 82 per cent agreement between three coders, which is above the level suggested by Miles and Huberman (1994).

## Results

### Episodes of Novelty in the Tourism Experiences

The findings from analysis of the novelty episodes revealed that there are two broad contexts where people experience novelty while traveling: a whole trip (trip-related) or an explicit event during travel (event-related). One main difference of these two contexts is the amount of detail remembered. Trip related novelty was often associated with first time travel experiences, such as “*I'm going to talk about my trip to Cuba first. It was a first trip to that region. It was a last-minute decision….” [RES_17]*. In a contrast, event-related novelty focused on a situation within a trip “*In Salerno we met a wandering artist with a dog. He was two meters tall, very skinny and spoke all European languages, including Russian.”* [RES_8].

Episodes involving novelty were identified as “*memorable”* [RES_24]*, “unforgettable”* [RES_15] and “*happening for the first time”* [RES_14]. According to participants responses, novelty of the tourism experiences is something that they had “*never experienced before*” [RES_10], which happened “*unexpectedly”* [RES_25] and was “*far from ordinary”* [RES_3]. Novelty for participants was described as a sense of being in an “*unfamiliar*” [RES_18] or a “*different*” [RES_3] environment. Novelty was associated with either an “*unforgettable trip* ***from beginning to end***” [RES_8] or a particularly “***significant moment”***within a trip [RES_2]. For instance, a participant mentioned that “*this trip was* ***the first time****when I was traveling for so long*,” highlighting the unusual **duration** of the trip that she “*never previously experienced”* [RES_14]. Related to a novelty associated with a whole trip, [RES_22] noted that “*visiting an Asian country for* ***the first time****in my life*” made his travel experience “*memorable*,” largely due to culture shock. In contrast to a novelty of a whole trip, [RES_12] highlights a particular event within a trip to Andorra when “*it was first time in my life I saw the mountains of the enormous scale.”* Whereas [RES_16] pinpointed an exact moment that made the trip novel “***remembering*** *every second of that* ***moment****when I saw the Eiffel tower, which I was* ***dreaming*** *about*.”

### Factors Influencing Novelty in Tourism

**Dreams** and **desires** have been identified as a significant theme in reported episodes of tourism novelty. A trip to Egypt to see the Pyramids and the Sphinx for [RES_24] “*was a childhood* ***dream***” and once achieved, he found this novel experience “*significant”* and “*more memorable”* than other trips. Elaborating on how he felt after a first-time trip to Byron Bay, Australia to learn how to surf, [RES_22] reported that “*my* ***dream****came true”* and that his “*next trip would be somewhere where I can surf* .” A “*childhood* ***dream***” for [RES_19] was go on a cruise and he reported that the “*fact that I'm going to a cruise made me feel* ***so excited****!*” Another respondent reported overcoming an obstacle to achieve her dream to see the Great Wall of China. “*I always had a* ***desire****to visit the Great Wall of China, but I was* ***scared****of the crowds. The pictures of thousands of tourists climbing the Wall was stopping me for* ***achieving my dream***” [RES_2]. Once achieved, she said the trip “*has overcome my expectations”* and now she thinks it was one of the “*peak experiences'* in her life, and unexpectantly there was *a lack of people which meant we had the place all to ourselves!*” [RES_4] mentioned that her trip to Lake Baikal in Russia is the one she “remember the most” because her future husband “*really wanted it to happen*' and “*was* ***dreaming****about it*.”

**Goals** and **intentions** to visit a particular novel destination were another significant theme. To illustrate this, [RES_5] mentioned that a “*trip to Bali was in a list of the life* ***goals****I wants to achieve before I die*.” Life/ travel goals and intentions category appears as something that this participant “*really want it to happen*” [RES_25]. A participant identified her first visit to Xian as “*unforgettable*,” and that she was “*prepared to go”* because “*it was* ***my goal***…* and I made a list of the Xian sights that I really want to see and just follow the list*” [RES_14]. One of the participants mentioned they “*really wanted to visit Japan*,” but also that they wanted to go “*everywhere* ***where I haven't been*** ***yet***” [RES_1].

**Recollections of novelty of an event** were described by respondents with **richer contextual details** compared to a **novelty of the whole trip**. For instance, while reconstructing a trip to China [RES_2] mentioned they still remembered an exact phrase her friend told her on the Great Wall of China—“*if you shared* ***a special moment*** *like this with someone, you are bonded together for the rest of your life*.” While reconstructing “*a moment* ***remembered the most***” [RES_5] mentioned that “*the famous Norway's Trollfjord is* ***1000 meters high****and you have to walk for* ***11 km****to reach the peak*.” A participant describing event-related novelty reported being able to “***remember***…* every second of* ***that moment”***[RES_16], which they will “***never*** ***forget***” [RES_12].

### Outcomes of Novelty in the Tourism Experiences

Novel experiences described by respondents had a **strong emotional component**, both pleasurable and unpleasurable. First in life experiences were found to possess a highly **sentimental tone**. For instance, [RES_8] described her first hitchhiking trip with “*the sky became a roof over your head, and the road becomes a thread to home*.” [RES_11] stated that her **“*first trip****to America was like* ***the first****love, that cannot be overshadowed by anything.”* [RES_12] the moment when she saw mountains for the first time in life: “*still have a feeling that a part of me is still there – in Andorra's mountains.”* Another participant [RES_22] mentioned that the experience of surfing on his first trip to Byron Bay in Australia was “*like a good coffee – it takes time and then you are finding yourself addicted to it*.”

Participants described novel experiences as having embedded positive emotions and feeling a “*sense of absolute freedom*” [RES_8], an ability to “*break free”* [RES_4] and a sense of being “*lost in time*” [RES_2]. One of the participants mentioned that seeing Mongolian yurts for the first time was: “***so exciting!***” [RES_14**]**. Some unforgettable experiences shared by respondents were very positive. For instance, [RES_17] said she “*was* ***happy****almost every second of that trip.”* Along with “*being happy*,” [RES_6] mentioned that he experienced happiness when “*share[ing] these emotions and experiences*” with other people.

**Surprise** was also identified as accompanying novelty in the tourism experiences. For instance [RES_10] reported: “*It was my first* ***overseas trip***…*I remember, I asked a local person in a shop: how much (in English)? and he answered me in Chinese. I was so* ***surprised****! It seems that everyone around the world nowadays speaks in Mandarin*.” [RES_1] said his first yacht trip “***delightful****and* ***happy****atmosphere.”* [RES_15] described a **surprising event** during the unforgettable trip as a very “*touching moment.”*

Episodes of tourism novelty were associated with **nostalgic** feelings. Participants “*wanted to come back*” [RES_14] to places they had visited for “*the first time”* and where they “*felt so good*” [RES_20]. [RES_14] said: “*I* ***want to go back****to certain places in Italy and to see certain people.… I want to go back to the same hotel, I want to go back to the same sea shore, to the same cafe. I feel so* ***nostalgic***.”

Respondents recalled both **positive and negative unexpected events** during travels. Negative novel experiences were described as “*unlucky*,” “*unsuccessful*” [RES_16], and as “*far from what was expecting*” [RES_17]. While describing her first banana boat experience [RES_10] mentioned being “***scared***” and “***horrified***” as she can't swim, and nobody explained that she has to do it. Interestingly, one of the respondents, **re-evaluated** a **negative** first-time travel experience with her boyfriend **as positive**, even though their baggage was stolen, and the experience was “*difficult*” and not “*impressive at all*.” This respondent mentioned that “*challenges…were really critical for our future relationships…Difficulties make couple stay together or separate. We went through this together as a team… Tenerife by itself didn't impressed me at all”* [RES_17].

**Experience sharing** emerged as another significant theme. According to participants, sharing unforgettable travel experiences with other people makes an experience “*have sense*” [RES_6], “*meaningful*” [RES_23], and “*memorable*” [RES_9]. Sharing an emotional event in a trip to new destination was extremely important for one of the participants who mentioned that **happiness** from visiting Malaysia came from an ability to “*share these emotions and experiences with others”* [RES_11].

Another theme was labeled **vivid and detailed memories**. Thus [RES_2] described details of a visit to the Great Wall of China, including words she still remembered, for instance “*he called me ‘sweetie'*, ***no one****called me like this* ***before****.”* A respondent recalled her peak experience during a trip 10 years before: “*I will* ***never forget****Italy. In Salerno we met a wandering artist with a dog. He was two meters tall, very skinny and spoke all European languages, including Russian. He was from Slovenia and his name was Ivan, and the dog's name was Doris…”* [RES_8].

## Discussion

This research explore the intersection between novelty and the memorability of tourism experiences. Data included recalled and reconstructed tourism experiences, with analysis designed to focus explicitly on the episodes of novelty during travels, factors influencing novelty, as well as the outcomes of novelty in the tourism experiences. This research to identified the antecedents and consequent conditions of novelty in the tourism experiences. Additionally, in-depth investigation on the role of novelty in the process of cognitive appraisal was considered. The following section connects the findings in this research with tourism and cognitive psychology. Findings of this research bridge a perceived gaps in existing literature on MTEs, Antecedent and consequent conditions of novelty in tourism are integrated into a conceptual framework of cognitive appraisal of novelty in MTEs (Figure 1).

### Reconstruction of the Episodes of Novelty in the Tourism Experiences

Results indicate that novelty influences how spatial, temporal, contextual, and emotional details of tourism experiences are remembered and reconstructed. Novelty influences the reconstruction and re-evaluation of experiences, which is defined as *memorability*. A connection between experiencing novelty and the ability to recall novelty episodes with more accuracy, particularly for specific events was identified. Episodes of novelty were identified to be focused on recollections of an entire trip (*trip-related novelty*) or narrowed in on a single event (*event-related novelty*) within a trip. The main criteria of difference between these two contexts is the amount of detail recalled and reconstructed. Trip-related novelty was found to be associated with comparably low levels of emotional and contextual details during recollection and reconstruction. The reconstruction of trip-related novelty in tourism included broader spatial and temporal details of the trip, such as novel geographical location, new duration of the trip or unusual distance to the destination, while event-related novelty focused on a specific situation within a trip. Event-related novel experiences within a trip were recollected by respondents with richer contextual and emotional details compared to a novelty of the whole trip.

Recall of detailed trip and event related experiences was noted and explained through increased attention identified by respondents when recalling MTEs. This is consistent with studies in the cognitive psychology and neuroscience literature on autobiographical memory (Radvansky and Zacks, 2011), memory retrieval (Tulving et al., 1996), flashbulb memories (Winograd and Neisser, 1992), as well as the studies on stimulus novelty and orienting response (Kishiyama and Yonelinas, 2003). This research confirmed that novelty facilitates recall of the tourism experience with more accuracy through attention since those unusual and new activities to which participants paid attention are more likely to be remembered. Thus, extant studies on autobiographical and flashbulb memories explained that novelty linked to intense emotions and are more likely to be remembered.

### Antecedents of Novelty in the Tourism Experiences

Thinking about the past (*retrospection*) and future (*prospection*) will be used as the lens to identify and critically analyze the antecedents and consequences of novelty in tourism experiences. This research found that retrospective antecedents are involved the process of recollection, while prospective antecedents on novelty in the tourism experiences associated with anticipation of future events as reflected on the model of cognitive appraisal of novelty in MTEs (see Figure 1).

Retrospective thinking about past travel influences how novelty in tourism is discussed. *Retrospection* involves detailed reconstruction of an event from memory with an accompanying sense of self (Shiffman et al., 1997; Miller et al., 2010; Sadeh et al., 2014). This research indicates the process of recollection of memories of prior travel experiences is associated with reconstructed emotion as well as memories consistent with CAT.

Utilizing retrospective thinking to analyze antecedent conditions of novelty in tourism, this research identified that *prior experiences* can be interpreted as a broader retrospective antecedent of novelty in tourism, which could be further explained through the concepts of *mental schema, emotions*, and *memories about past trips*, including *nostalgia*. Prior experience identified as an important condition of novelty as it forms traces of similar experiences in the past which allows participants to experience novelty in the present and allows potential recollection and reconstruction in the future.

This research has also identified that apart from recollection, experience of novelty in tourism also involves the process of anticipation of the future events which links novelty to the concept of prospection. *Prospection* refers to a prototypical biased vision of the future toward pleasurable experiences, and grounded in schemas, stereotypes, personal goals, and other mental representations of what person is typically like (Kane et al., 2012). Concomitantly, prospection is similar to affective forecasting or imagining what our future travels will be like under various scenarios (Skavronskaya et al., 2017a). Overall, prospective thinking about the antecedents of MTEs connects novelty with the concepts such as goals, expectations, dreams, and fantasies as reflected on the proposed Figure 1.

The concepts of anticipation and prospection enhances the conceptual understanding of the integral role of fantasies and goals in the cognitive appraisal of novelty in tourism. In this research respondents recalled travel episodes from the past, including the recollection of the feelings prior the trip which involves the excitement. In turn, anticipation of future travel was intertwined with concepts such as interest and goals for the trip which in turn stimulated obsessive thinking about future travel, which is fantasy (Le et al., 2019). Following this cognitive process, during an actual trip, a person had stronger emotional response due to achievement of the trip-related goals.

Overall, antecedents of novelty in tourism included retrospective and prospective components. The prospective component involves anticipation of future events, expectations, and fantasies about future travels. Stronger goals mean that greater expectations and stronger emotion and memorability when novelty related to a goal is experienced. Recollection of novelty requires recreation of memories and of past experiences. These memories of past events are stronger and hence easier to recall when an event involves novelty.

### Consequences of Novelty in the Tourism Experiences

The consequences of novelty in tourism can be divided into immediate and long-term. To acknowledge the difference between long-term and immediate consequences of novelty in tourism, relevant section has been included on the conceptual framework of cognitive appraisal of novelty in MTEs (see Figure 1).

Immediate consequences of novelty in the tourism experiences include intense emotions and feelings, and relay memories related to the novel experiences with greater potential to be transferred into long-term memory. Neuropsychological studies suggest that information about novel experiences is encoded and transferred into long-term memory (Tulving et al., 1994; Tulving and Kroll, 1995). As has been previously mentioned, novelty is also connected to enhanced attention. Greater attention to novel experiences increases the amount information that is encoded and stored in memory (Dijksterhuis and Aarts, 2010; Van Kesteren et al., 2012). Short-term memory is able to store information for only a short period of time and with limited capacity, whereas long-term memory has greater capacity and duration (Atkinson and Shiffrin, 1968). Novelty is considered a necessary condition for encoding information in long-term memory (Tulving and Kroll, 1995) and enhances the capacity for interactions between the different memory systems (Henson and Gagnepain, 2010).

Long-term consequences of novelty include enhanced memorability and ease of recall (reminiscence) (Webster, 1994; Tung and Ritchie, 2011b). Reminiscence or recall and reprocessing of past events novelty leads to re-evaluation and the change of meaning associated with the event. An example may be the re-evaluation of an unexpected and unpleasant travel experiences (e.g., stolen luggage) as positive if person's related goals were achieved. Thus, re-evaluation involved reassessment based on the consistency of the particular travel experiences with the persons' goals. This finding is consistent with the previous tourism literature on role of emotions and goals in cognitive appraisal process in tourism (Hosany, 2012; Ma et al., 2017; Campos et al., 2018). Goals can also guide behavior by direction of attention to salient objects (Dijksterhuis and Aarts, 2010). Novelty of an object salient to a goal captures attention.

This research found that intense emptions is a consequent condition of novelty in recalled events. Previous studies recognize that emotions related to the memorability of the tourism experiences (Bastiaansen et al., 2019). Findings of the present research indicate that novelty may lead to positive as well as negative emotions in the tourism experiences (Kim J. H et al., 2012; Lin et al., 2014; Wang et al., 2017). Thus, novelty is independent of emotional valence (Förster et al., 2010). Emotional valence is instead determined by appraisals of goal congruence and novelty associated with goal congruent experience will result in pleasurable emotions (Mitas and Bastiaansen, 2018).

Novel pleasurable events were often associated freedom (Moore et al., 1995) surprise, happiness, excitement, and delight (Ma et al., 2017). Interestingly, it has been found that positive vivid memories of novel travel experiences are related to nostalgia and intention to revisit a destination (Hwang and Hyun, 2013; Bergs et al., 2019). Nostalgia is included in the retrospective part of the conceptual framework (Figure 1) and the relationship about the effect of novelty on nostalgia represents a prominent area for future scholarly inquiry. Participants also shared negatively remembered tourism experiences involving novelty, as a result of unexpected illness, injury and other losses, or heightened periods of emotional stress. As a result of unpleasant travel experiences, participants feel disappointment, unexpectedness, fear, horror (Petzer et al., 2012; Weaver et al., 2018).

## Conclusion, Implications, and Future Research

Novelty is a cognitive appraisal dimension with the capacity to enhance attention and emotions and hence create lasting vivid memories. Novelty influences the intensity of emotions, stronger memory and therefore affects the ease of the experience reconstruction (memorability) and reminiscence. This research developed a conceptual model that explores the role of novelty as a cognitive appraisal dimension in the formation of MTEs through the elicitation of emotions, but also associations with cognitive processes such as goals, attention, and expectations through retrospective and prospective approach to imagined and recalled tourism experiences (see Figure 1).

This research contributes to the tourism field by exploring how theory from cognitive psychology can be utilized to provide a more nuanced understanding of the tourism experience and vice-versa (Tung and Ritchie, 2011a; Kim and Brown, 2012; Kim J. H et al., 2012; Kim K. et al., 2012; Pearce and Packer, 2013; Torland et al., 2015; Scott et al., 2017; Moyle et al., 2019; Huang et al., 2020). Specifically, this research adds value to the studies on mental processes associated with tourism experiences (Ma et al., 2013, 2017; Campos et al., 2016, 2018; Skavronskaya et al., 2019a) and contributes to a better understanding of the role of novelty both an antecedent to, and consequence of, MTEs (Chandralal and Valenzuela, 2015; Mitas and Bastiaansen, 2018; Skavronskaya et al., 2019a).

While focused on tourism, this research is also designed to stimulate debate in cognitive psychology surrounding the efficacy of tourism for advancing knowledge within the subdiscipline. Traditional cognitive psychology uses experimental approaches, often in a laboratory setting and these methods and techniques are available to study internal processes associated with tourism and travels (Li et al., 2015; Hadinejad et al., 2019; Scott, 2020). However, tourism provides an opportunity outside of laboratory settings where cognitive theories can be applied. Understanding of the mental processes that underpins tourism experiences help better understanding of tourists' behavior.

The value of novelty for tourism industry is demonstrated through enhanced tourism experience design. For instance, by implementing novel, unexpected and surprising components for designing tourism experiences and marketing campaigns, tourism practitioners can increase re-visitation, form loyalty and generate tourist's satisfaction (Chen and Rahman, 2018; Kim, 2018). Understanding novelty in tourism can increase emotional engagement with tourists at the destination (Michalkó et al., 2015), presenting an opportunity for entrepreneurs to operationalize novelty to engage tourists at a deep and profound level. Additionally, due to the rapid expansion of cutting-edge technologies there is an avenue to further explore, interpret, and implement novelty to MTEs through technologies, such as virtual and augmented reality (Bec et al., 2019).

Further conceptualization of novelty in tourism is required as a number of theoretical gaps has been identified. For instance, in the extant tourism literature on emotional and MTEs (Petrick, 2002; Tung and Ritchie, 2011a; Ma et al., 2017) the words novelty, unexpectedness and surprise are often used interchangeably, and the concepts are not distinguished from one another. However, the concepts have been expensively explained in cognitive and neuroscience (Plutchik, 1980; Meyer et al., 1991; Barto et al., 2013). Therefore, there is a need to distinguish between novel, surprising, and unexpected tourism experiences. Furthermore, extant tourism studies are largely focused on novelty seeking behavior (Kim and Kim, 2015), rather than novelty as appraisal dimension. Thus, the differences between novelty and novelty-seeking in tourism experiences should be examined further. Due to the hedonic nature of the tourism experiences, most studies on the role of novelty in positively remembered tourism experiences (Ma et al., 2013, 2017; Mitas and Bastiaansen, 2018). The role of novelty as an appraisal dimension in tourism experiences perceived as negative is not addressed in the tourism literature.

## Data Availability Statement

The datasets generated for this study are available on request to the corresponding author.

## Ethics Statement

The studies involving human participants were reviewed and approved by Griffith University—2017/751. The patients/participants provided their written informed consent to participate in this study. Written informed consent was obtained from the individual(s) for the publication of any potentially identifiable images or data included in this article.

## Author Contributions

The outcomes of this research are based on LS PhD dissertation titled (*Novelty in Memorable Tourism Experiences*). LS played a key role in conceptualization, data collection, analysis, and drafting of the manuscript. BM and NS played a critical role in the conceptualization of the research, with further guidance provided by BM focused on the method and analysis. All parties contributed to the writing and reviewed the final draft several times prior to submission.

### Conflict of Interest

The authors declare that the research was conducted in the absence of any commercial or financial relationships that could be construed as a potential conflict of interest.
